# Machine Learning Predicts 30-Day Outcome among Acute Myeloid Leukemia Patients: A Single-Center, Retrospective, Cohort Study

**DOI:** 10.3390/jcm12185940

**Published:** 2023-09-13

**Authors:** Howon Lee, Jay Ho Han, Jae Kwon Kim, Jaeeun Yoo, Jae-Ho Yoon, Byung Sik Cho, Hee-Je Kim, Jihyang Lim, Dong Wook Jekarl, Yonggoo Kim

**Affiliations:** 1 Department of Laboratory Medicine, Yeouido St. Mary’s Hospital, College of Medicine, The Catholic University of Korea, Seoul 07345, Republic of Korea; 2 Department of Laboratory Medicine, Seoul St. Mary’s Hospital, College of Medicine, The Catholic University of Korea, Seoul 06591, Republic of Korea; 3 Department of Laboratory Medicine, Incheon St. Mary’s Hospital, College of Medicine, The Catholic University of Korea, Incheon 21431, Republic of Korea; focused@catholic.ac.kr; 4 Division of Hematology, Department of Internal Medicine, Seoul St. Mary’s Hospital, College of Medicine, The Catholic University of Korea, Seoul 06591, Republic of Korea; 5 Department of Laboratory Medicine, Eunpyeong St. Mary’s Hospital, College of Medicine, The Catholic University of Korea, Seoul 03312, Republic of Korea; 6 Research and Development Institute for In Vitro Diagnostic Medical Devices, College of Medicine, The Catholic University of Korea, Seoul 06591, Republic of Korea

**Keywords:** acute myeloid leukemia, early death, machine learning, decision tree, classification, hemorrhage, fibrinogen, infection

## Abstract

Acute myeloid leukemia (AML) is a clinical emergency requiring treatment and results in high 30-day (D30) mortality. In this study, the prediction of D30 survival was studied using a machine learning (ML) method. The total cohort consisted of 1700 survivors and 130 non-survivors at D30. Eight clinical and 42 laboratory variables were collected at the time of diagnosis by pathology. Among them, six variables were selected by a feature selection method: induction chemotherapy (CTx), hemorrhage, infection, C-reactive protein, blood urea nitrogen, and lactate dehydrogenase. Clinical and laboratory data were entered into the training model for D30 survival prediction, followed by testing. Among the tested ML algorithms, the decision tree (DT) algorithm showed higher accuracy, the highest sensitivity, and specificity values (95% CI) of 90.6% (0.918–0.951), 70.4% (0.885–0.924), and 92.1% (0.885–0.924), respectively. DT classified patients into eight specific groups with distinct features. Group 1 with CTx showed a favorable outcome with a survival rate of 97.8% (1469/1502). Group 6, with hemorrhage and the lowest fibrinogen level at diagnosis, showed the worst survival rate of 45.5% (25/55) and 20.5 days. Prediction of D30 survival among AML patients by classification of patients with DT showed distinct features that might support clinical decision-making.

## 1. Introduction

Acute myeloid leukemia (AML) is a clonal proliferative disorder caused by abnormal cytogenetic abnormalities or mutations [1,2]. The global incidence and mortality of leukemia were 5.4 and 3.3 per 100,000, respectively, in 2020 [3]. Around 1.04% (20,380/1,958,130) of total patients were newly diagnosed with AML in 2023. The five-year survival rate showed increasing tendencies, changing from 34% to 48% and 66% in the United States during 1975–1977, 1995–1997, and 2012–2018, respectively [4].

Although survival rates have been increasing, AML is a medical emergency requiring prompt treatment after diagnosis [5]. Before induction chemotherapy, cytoreduction by pharmacologic agents or mechanical removal of leukemic cells by leukapheresis could be performed as a bridging therapy [5,6]. Chemotherapy (CTx) measures include standard 3 + 7 therapy (Standard CTx), FLAG (fludarabine, anthracycline, G-CSF), hypomethylating agents (HMA), low-dose cytarabine (LDAR), mitoxantrone, etoposide, and cytarabine combination CTx (MEC), which could be applied to patients considering age, organ dysfunction, and performance status [7,8,9,10]. Targeted therapy based on tumor neoantigens with respect to genetic mutations is under current development and is entering the clinical field [11,12,13,14,15]. Hematopoietic stem cell transplantation could be performed for eligible patients and could be a curative measure.

Initial presentation of AML could show a wide variation from an asymptomatic state to the patient requiring intensive care [12]. Severe symptoms requiring emergent intervention related to AML are as follows: tumor lysis syndrome (TLS), bone marrow dysfunction, which induces anemia, infection, and bleeding, and leukostasis symptoms due to hyperleukocytosis, respectively. These clinical features may threaten a patient’s early 30-day survival.

Although treatment modalities have been introduced for AML treatment, early death comprises 5–11% of AML patients [16,17]. To predict patients at high risk for D30 death, various factors are suggested. Mendes et al. suggested that age, Gram-negative bacterial infection, monocytic AML, increased C-reactive protein (CRP), and adverse genetic risks were associated with an increased risk of 60-day mortality [18]. Sasaki et al. suggested the following prognostic factors for D30 death after intensive chemotherapy (CTx): age, ECOG performance status, infection at diagnosis (pneumonia), cytogenetics (complex karyotype), total bilirubin, and creatinine and uric acid [16].

Advances in machine learning (ML) methods are increasingly applied for treatment selection and disease subclassification by integrating data or predicting prognosis for personalized medicine in hematologic malignancies [19,20,21]. Prediction of mortality was performed for patients admitted to the ICU with acute renal replacement therapy and for patients admitted to the ICU using time series data associated with vital signs or organ function [22]. In hematology, ML has been applied to the diagnosis of subtypes of AML, minimal residual disease in AML, and myelodysplastic syndrome. Outcome prediction was studied after hematopoietic stem cell transplantation or graft versus host disease after transplantation [23]. Park et al. used XGBoost or the K-NN algorithm to predict the CTx response of multiple myeloma, which could lead to personalized medicine [24]. Awada et al. used the random forest for the subclassification of primary and secondary AML by integrating genomic and clinical data for big data interpretation [25].

In this study, the D30 survival rate was analyzed to predict associated factors that might support clinical decisions for treatment. We focused on the decision tree (DT) to classify the high-risk group for D30 survival because DT was interpretable and showed the highest sensitivity.

## 2. Materials and Methods

**Figure 1:**
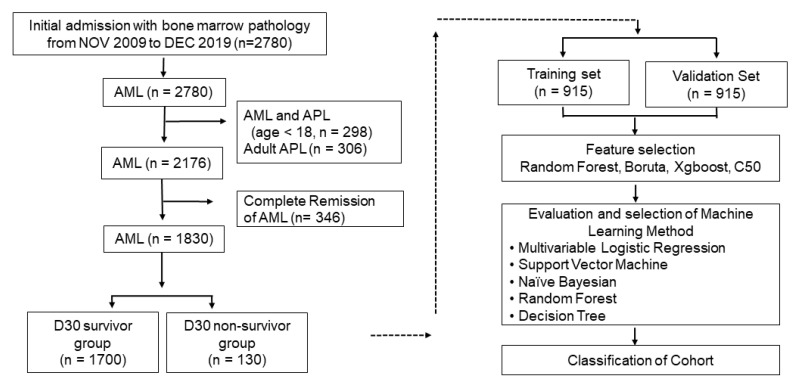
Flow chart and study summary for machine learning.

This was a single-center, retrospective cohort study that was approved by the Institutional Review Board of Seoul St. Mary’s Hospital, The Catholic University of Korea. To determine the D30 prognostic factors, an AML patient cohort was constructed using the Common Data Warehouse of The Catholic University of Korea from April 2009 to December 2019. Enrollment in this study included patients over the age of 18 who were initially admitted to the institution and underwent a bone marrow biopsy upon admission (Figure 1). The diagnosis of AML was based on the WHO fourth edition guidelines, and the ELN classification was based on the 2017 version [6,26,27].

Finally, 1830 patients were enrolled, comprising 1700 D30 survivors and 130 non-survivors. The demographic and clinical data are presented in Table 1. Laboratory data were collected at the time of the bone marrow biopsy and are listed in Table 2. A total of 50 variables were extracted, which included 8 clinical and 42 laboratory, or 7 categorical and 53 continuous variables (Table 2).

Data on demographics were complete for age, sex, and diagnosis, as were those on a complete blood count. Blood chemistry results were missing for up to 15% of cases and were analyzed as median values. Hematocrit was included in the study, while red blood cells and hemoglobin were omitted for machine learning based on their correlation coefficients of less than 0.90. To reduce the size of the comorbidity subgroup, logistic regression analysis was performed; comorbidity was classified as renal disease and others (Supplementary Table S1). Continuous data were scaled by subtracting the mean divided by the standard deviation.

Data were divided into a training data set (*n* = 915) and a test data set (*n* = 915) by random sampling. To determine the D30 prognostic factors, various machine learning algorithms were performed using the R package Caret and other associated packages [28].

Feature selection was performed using the recursive feature elimination method (RFE). Feature selection is the process of selecting or excluding irrelevant variables for constructing the model. In this study, the random forest and randomForest R packages were used to exclude unimportant variables [28,29]. In addition, to validate the selected features, Boruta, Xgboost, and the C50 algorithm were selected. Boruta ranks and selects features based on the random forest algorithm. Xgboost, or extreme gradient boosting, uses the CART ensemble method. The Xgboost algorithm is based on gradient boosting, which is performed by finding a function that minimizes the gap between data and calculated data. Xgboost has additional parameters that could prevent overfitting. C50 is an algorithm to generate a decision tree that uses information entropy to generate a homogenous group repeatedly that results in a tree structure [21,22,23,24,25,26,27,28,29,30,31,32]. As the dataset was relatively small for applying the ML method, it was divided into training and test sets during the 10-fold cross-validation method, which was applied to minimize error and construct the optimal model.

Multivariable logistic regression (MLR) was performed to identify variables that could predict binary outcome probabilities. Variables of statistical significance in the univariable analysis were entered into the MLR analysis. A revised MLR analysis was performed, followed by a model revision using variables selected by the feature selection method.

The support vector machine (SVM) method was used for the classification of D30 survivors and D30 non-survivors by finding the hyperplane with the largest margin between the two groups. This plane, or hyperplane, gives the maximum separation of the data between two classes. Support vectors are data points that influence the plane or hyperplane. These support vectors are used to maximize the distance between planes, or hyperplanes, which results in the classification of two classes. The SVM model was constructed and revised based on the grid search algorithm [33].

Random forest (RF) uses the bootstrapping method, which samples with replacement multiple times. Each of the sampled groups is trained independently to produce a decision tree. Therefore, multiple decision trees are formed. By averaging these results, consensus predictions are generated. By not considering strong predictors, RF could generate heterogeneous trees, which could lower variance and lead to more reliable results. The randomForest R package is additionally used for analysis [28,34].

The Naive Bayesian algorithm (NB) is based on Bayes’ theorem and assumes that the variables are conditionally independent. The Bayesian network model was subjected to kernel density estimation for classification. The naive Bayesian and e1071 R packages were used for analysis [35].

A decision tree (DT) splits the data multiple times to achieve a homogeneous group. Splitting of the data results in homogenous groups, which could be defined by the Gini index or entropy. The lower value of the Gini index, or entropy, denotes a homogenous group. The growth of the tree or splitting of the data goes on until the terminal nodes are reached. The CART algorithm in the R package rpart was used, followed by the e1071 package for tuning the model [36]. DT starts from a root node that represents the data in the more specified uniform group until the terminal root node is reached. Pruning a tree once will result in two terminal nodes, twice in four maximum terminal nodes, and three times in a maximum of eight nodes. The advantage of DT is that the interpretability of the data and graphical display are possible. The disadvantages are lower accuracy compared to other models and the fact that trees are non-robust, indicating that small changes could alter the structure of the tree.

## 3. Results

The use of feature selection algorithms resulted in the recommendation of six features as the optimal number. These features were induction, hemorrhage, infection, blood urea nitrogen (BUN), direct bilirubin, and lactate dehydrogenase (LDH) by the RFE method (Supplementary Tables S2 and S3). Features selected by other methods are listed in Supplementary Table S4. C-reactive protein (CRP) was selected instead of direct bilirubin.

**Figure 2:**
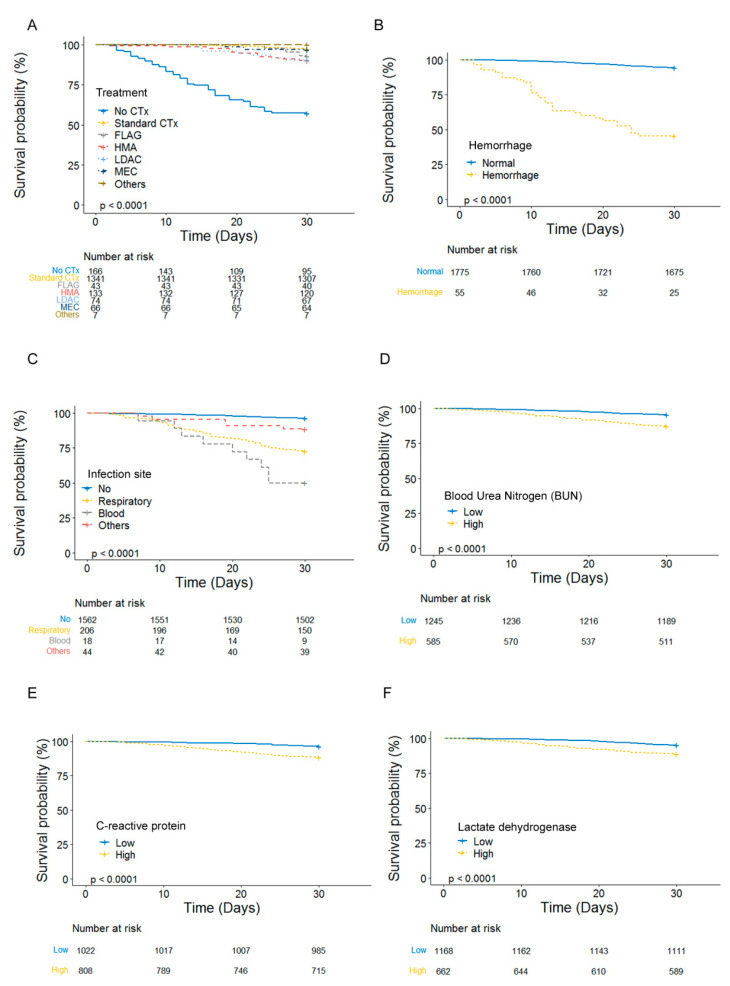
Six variables were selected by feature selection. (**A**) Chemotherapy, (**B**) Hemorrhage, (**C**) Infection, (**D**) Blood urea nitrogen (BUN), (**E**) C-reactive protein, and (**F**) Lactate dehydrogenase.

This choice was made because it performed better in subsequent machine learning experiments. To determine the clinical importance of these variables, a D30 survival analysis was performed (Figure 2). Patients who could not be treated with chemotherapy showed lower mean D30 survival compared to standard chemotherapy (22.7 d vs. 29.7 d). Patients with hemorrhage showed a worse mean D30 survival compared to patients without hemorrhage (20.5 d vs. 29.3 d). Bloodstream microbial infection was associated with a worse mean D30 survival, followed by respiratory infection, other infections, and no infection (24.1 d, 26.1 d, 28.4 d, 29.5 d). For laboratory variables, the groups with higher BUN showed a worse mean D30 survival compared to the lower group (28.2 d vs. 29.4 d). The high direct bilirubin and LHD group showed a worse mean D30 survival time compared to the group with lower values (28.3 d vs. 29.4 d) (Figure 1, Supplementary Table S5).

### 3.1. D30 Prediction by ML

Univariable logistic regression was performed and resulted in 32 variables entering multivariable logistic regression analysis (Supplementary Table S6). Induction, hemorrhage, infection, bilirubin, and direct bilirubin showed statistical significance and were entered into the multivariable model (Supplementary Tables S7 and S8). The performance of the model using these variables showed an accuracy rate (95% confidence interval [CI]) of 89.5% (0.8734–0.9142), which decreased to 87.8% (0.8558–0.8991) when using the six variables from feature selection (Table 3, Supplementary Table S9).

SVM showed an accuracy rate (95% CI) of 93.3% (0.9152–0.9486) with 0% sensitivity. The revised model using six features showed an accuracy rate (95% CI) of 91.1% (0.8901–0.9281) with a sensitivity rate of 14.7%. Tuning based on the grid search algorithm with a cost value of 100 and a gamma value of 0.01 resulted in an accuracy rate (95% CI) of 93.3% (0.9152, 0.9486) with a decreased sensitivity rate of 0%.

The NB showed an accuracy rate (95% CI) of 89.7% (0.8687–9.102) with 34.4% sensitivity. The revised model using six features showed an accuracy rate (95% CI) of 91.4% (0.8936–0.9311) with a sensitivity rate of 37.7%.

RF showed the highest accuracy rate among the tested ML algorithms, which was 94.7%, with a 95% CI of 0.9310–0.9611. After entering the six features into the revised model, a slightly decreased accuracy (95% CI) of 93.7% (0.9200–0.9525) was obtained. However, sensitivity was in the range of 20–30%.

### 3.2. Decision Tree (DT)

**Figure 3:**
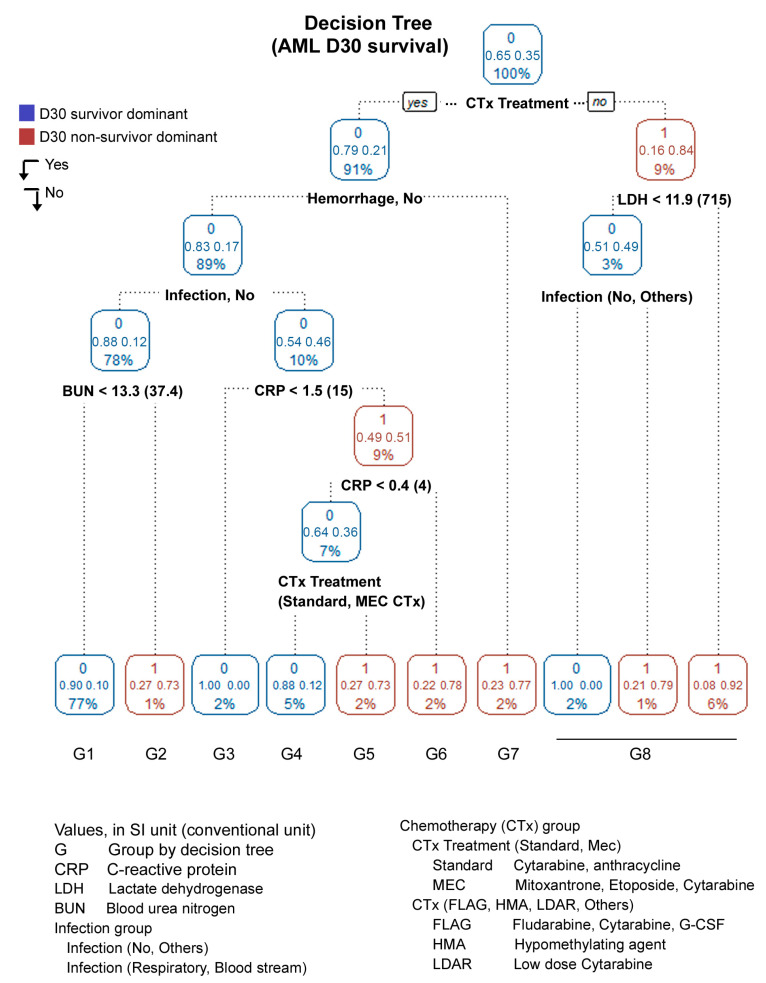
Decision tree for prediction of D30 survival among AML.

As the DT revealed one of the highest sensitivities among the tested ML algorithms, we focused on it. The DT used the three variables of induction, LDH, and CRP to classify subgroups, showing an accuracy rate (95% CI) of 93.6% (0.9188–0.9515). The model was revised by entering the six features and showed an accuracy rate (95% CI) of 90.6% (0.8852–0.9241). Although the accuracy rate decreased compared to the initial model, the revised model showed an enhanced sensitivity rate of 70.4% compared to 29.5% of the initial DT model. Indeed, the sensitivity of the DT model was the highest among the tested ML algorithms. The classification tree using the six variables is plotted in Figure 3. Blue- or red-colored figures indicate the D30 survivor-dominant or D30 non-survivor-dominant groups, respectively. The root node started regardless of whether the patient received any kind of CTx or was treated due to the patient’s condition or other factors. The decision tree was classified into eight groups because there were only two patients in the group who were not treated with CTx and had low LDH.

### 3.3. Prognosis

**Figure 4:**
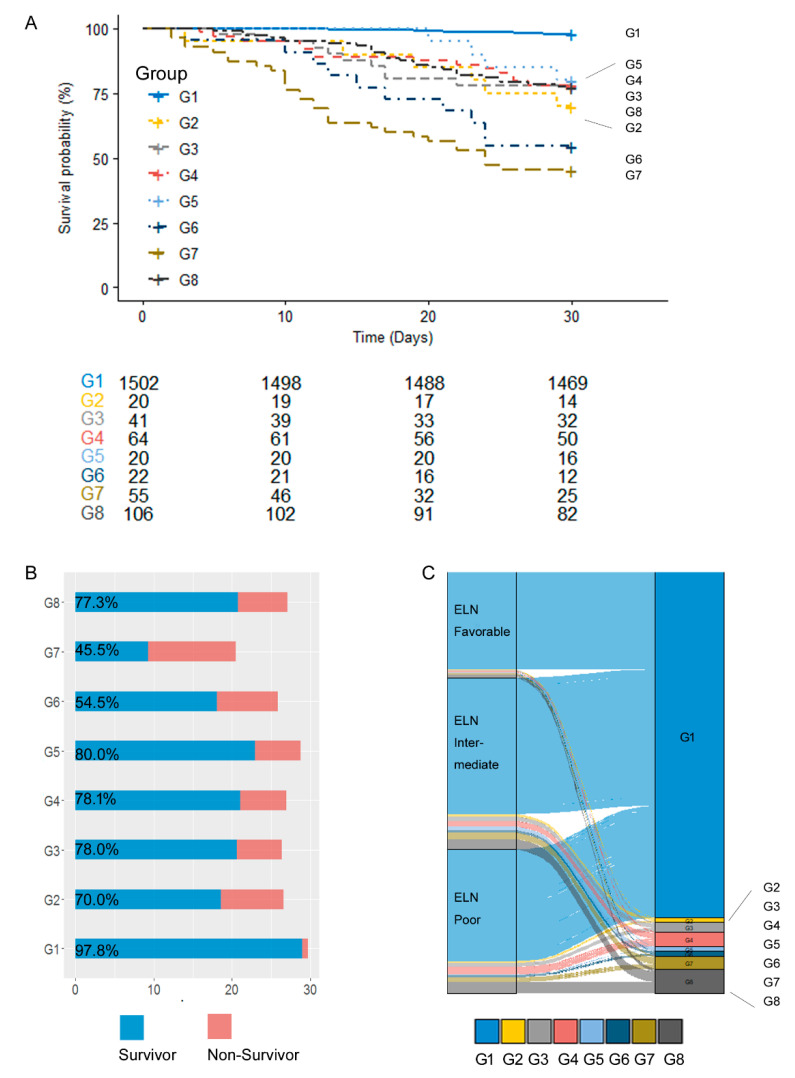
Survival analysis of classified groups from the decision tree. (**A**) survival curve of each group; (**B**) survival days and the ratio of survivors of the classified group; (**C**) flow diagram of ELN risk classification groupS allocated to G1 to G8.

The decision tree classified the patients into eight groups. Using the total cohort (*n* = 1830), further interrogation was performed to search for clinical significance (Figure 4A). Classified groups had distinct laboratory and clinical features compared to other groups (Supplementary Table S10).

D30 survival analysis was performed for eight groups. Mean survival days (d) and standard error are listed (Supplementary Table S11) along with pairwise comparison results (Supplementary Table S12). The survival analysis showed that G1 included 1502 patients, and G1 showed the highest survival ratio, with 97.8% of patients surviving during D30 (Figure 4B, Supplementary Figures S3 and S4). G2 showed 70% (14/20) survival and marked the highest aspartate aminotransferase (AST), creatinine, uric acid, and LDH levels. G3 to G6 could be clustered as an infection group. Among them, G3 showed the highest CRP, ESR, and glucose levels, along with a 78% (32/41) survival rate. G4 showed the highest platelet count. G5 showed the highest age and lymphocyte ratio but the lowest white blood cell count, CRP, AST, and LDH levels, resulting in an 80% (16/20) survival rate, which was the second highest. G6 showed the highest WBC count and the lowest lymphocyte ratio, marking the second-worst prognosis group. G7 included 55 patients who showed the worst outcome, and only 45.5% of them survived during D30. G7 showed the lowest fibrinogen level and the second lowest platelet count, but not prothrombin time (PT) or activated thromboplastin time (APTT). G8 was not treated for CTx and had the highest proportion of complex karyotyping results, which was 16% (17/106). The ELN 2017 risk group was allocated to eight groups, and it showed that all of the ELN risk groups were dispersed from G1 to G8 (Figure 4C). However, the ELN intermediate and poor prognostic group comprised a higher ratio of G2 to G8 compared to G1.

### 3.4. Prediction of Hemorrhage

Hemorrhage during the first 30 days after diagnosis was one of the critical factors for D30 survival. On multivariable logistic regression analysis, high BUN and alkaline phosphatase were associated with hemorrhage with an odds ratio of 1.48 (1.06–2.06) and 1.32 (1.00–1.75), respectively. Low fibrinogen and phosphorous concentrations were associated with hemorrhage with an odds ratio of 0.61 (0.41–0.92) and 0.62 (0.41–0.93), respectively (Supplementary Table S10).

## 4. Discussion

As the D30 prognostic factor predicts a short survival duration, infection at the time of admission, patient eligibility for intensive CTx or low-dose CTx, including HMA and LDAR, the presence of hemorrhage, and laboratory parameters like CRP, BUN, and LDH were important prognostic factors. Genetic abnormalities, including cytogenetics and ELN classification, were not important D30 predictive factors but were associated with complete response to CTx or 5-year survival [37].

Machine learning algorithms have been applied to clinical data for diagnosis, classification of disease, prognosis prediction, and prediction of treatment outcomes. In this study, most of the ML algorithms suffered from low sensitivity. This might be caused by a low positive rate, as the D30 of non-survivors was only 7.1% (130/1830). Among various ML algorithms, the decision tree (DT) was interpretable and could be applied to the clinical setting in this study. As there are various ML algorithms, the selection of the appropriate ML algorithm could be an important factor in the outcome. Consideration of the data and interpretability might be a factor in selecting the optimal algorithm. Overfitting has been an obstacle to applying ML algorithms to testing data. In this study, the ML algorithm was trained with 50 variables and then applied to a test dataset. To reduce overfitting, we used the six variables that were entered into the model for revision. As one of the purposes of this study was to reveal D30 prognostic factors and search for countermeasures, ML with high sensitivity was selected, which was provided by the DT algorithm.

In this study, the AML patient cohort was classified into eight different groups, each with distinct features compared to the other group. Among them, group 7 (G7), which could be characterized as a hemorrhage group, showed the worst outcome with only 45.5% (25/55) of survivors, with a mean survival days of 20.6 d. G6 showed the lowest fibrinogen level compared to other groups; indeed, hemorrhage was associated with lower fibrinogen or inorganic phosphorus by logistic regression analysis. Prevention of hemorrhage might enhance AML patients’ early survival. However, a recent randomized controlled trial of prophylactic tranexamic acid in patients with hematologic malignancies did not significantly reduce WHO grade ≥2 hemorrhage [38]. As patients with a platelet count lower than 10,000/uL for five days were enrolled in that study, additional analysis might result in a better outcome using prophylactic tranexamic acid. Enrolling patients with decreased fibrinogen might have resulted in different outcomes. Prophylactic replenishment of fibrinogen or phosphorus might support patient treatment and might result in a better D30 outcome.

Infection, especially pneumonia at the time of diagnosis, is a well-known factor in D30 prognostics. In addition, C-reactive protein showed a prognostic role and reflected an inflammatory state. In this study, patients with infection were classified as G3 to G6; among them, G6 showed the worst survival rate of 54.5% (12/22). G6 had the highest WBC count or leukemic cell burden, and males comprised 81.8% (18/22). In AML, blast or leukemic cells suppress the immune system to enhance clonal expansion and infection [39]. Treatment of infection, including pneumonia, among immune-suppressed AML patients might prolong D30 survival by becoming a candidate for CTx.

Treatment of infection other than antibiotics or anti-fungal agents might be considered and granulocyte transfusion could be a treatment option. Granulocyte transfusion is a treatment modality with an insufficient outcome result based on a randomized controlled trial. Granulocyte transfusions are usually regarded as a last resort for infection treatment in patients with deteriorating conditions [40]. Granulocyte transfusion is hindered by the production of anti-HLA antibodies, which might result in platelet refractoriness or a reduced survival probability for HSCT. In this study, Group 6 showed the second-worst outcome, with the highest leukemic burden. As the survival rate was 54.5%, we carefully suggest that early granulocyte transfusions might be a treatment option for patients in Group 6 [41]. Although the context was different, early granulocyte transfusion increased the survival rate among patients with neutropenia caused by multi-drug-resistant organisms [42]. Group 2 (G) showed organ dysfunction at the time of diagnosis and had the highest BUN, creatinine, and AST levels compared to other groups. Although CTx is the treatment of choice, careful consideration and follow-ups during CTx are requested for the G2 group.

A limitation of this study was that hemorrhage was not graded by WHO classification. The presence of hemorrhagewas recorded according to the electronic medical records, genetic mutation by a next-generation sequencing panel was not applied in the study, which might result in a D30 prognosis. Treatment, including leukapheresis, therapeutic cytoreduction, and transfusion after diagnosis, was considered and required independent study for early survival. Unlike genetic laboratory data, laboratory data on complete blood count and blood chemistry can vary dramatically based on the patient’s condition. The data used in this study were collected on the day of the bone marrow biopsy before any treatment other than leukapheresis was performed. The dataset used in this study was relatively small for applying the ML method, and further studies using additional datasets containing various regions and ethnicities are required.

The prediction of D30 survival among AML patients was performed using a decision tree. The D30 prognosis was based on induction chemotherapy, hemorrhage, and infection at the time of diagnosis and laboratory data, including blood urea nitrogen, C-reactive protein, and lactate dehydrogenase. The decision tree might support the clinical decision-making process, which should be validated with further studies.

## 5. Conclusions

The prediction of D30 survival among AML patients was performed using a decision tree. Induction chemotherapy, hemorrhage, and infection at the time of diagnosis, and laboratory data, including blood urea nitrogen, C-reactive protein, and lactate dehydrogenase, were used to predict the D30 prognosis. The decision tree might support the clinical decision-making process, which should be validated with further studies.

## Figures and Tables

**Table 1:** Baseline characteristics of AML cohort.

		D30 Survivor	D30 Non-Survivor	*p*
	*n*	*n* = 1700 (%)	*n* = 130 (%)	
Age	1830	50.6 ± 15.5	60.1 ± 14.2	<0.001 *
Sex	1830			0.084
Female		781 (45.9)	49 (37.7)	
Male		919 (54.1)	81 (62.3)	
Diagnosis ^a^	1830			<0.001 *
BCR/ABL		7 (0.4)	0 (0.0)	
CBFB_MYH11		66 (3.8)	3 (2.3)	
CEBPA		72 (4.2)	1 (0.7)	
DEK/NUP		12 (0.7)	0 (0.0)	
FLT3		151 (8.8)	17 (13.1)	
GATA2/MECOM		14 (0.8)	2 (1.5)	
KMT2A		63 (3.7)	4 (3.1)	
MRC		145 (8.5)	11 (8.4)	
NOS		594 (34.9)	54 (34.9)	
NPM1		130 (7.6)	3 (2.3)	
NPM1-FLT3		38 (2.2)	2 (1.5)	
RUNX1_RUNXT1		181 (10.6)	4 (3.1)	
TAML		11 (0.6)	2 (1.5)	
Secondary AML		34 (2.0)	5 (3.8)	
AML MRC		182 (10.7)	22 (16.9)	
Induction	1830			<0.001 *
No CTx		95 (5.6)	71 (54.6)	
Standard CTx		1307 (76.9)	34 (26.2)	
FLAG CTx		40 (2.4)	3 (2.3)	
HMA		120 (7.1)	13 (10)	
LDAC		67 (3.9)	7 (5.4)	
MEC		64 (3.8)	2 (1.5)	
Others		7 (0.4)	0 (0.0)	
Hemorrhage	1830			<0.001 *
No		1675 (98.5)	100 (76.9)	
Yes		25 (1.5)	30 (23.1)	
Infection				<0.001 *
No		1482 (88.6)	58 (45.3)	
Respiratory		146 (8.7)	56 (43.8)	
Bloodstream		8 (0.5)	9 (7.0)	
Others		36 (2.2)	5 (3.9)	
Cytogenetics	1626			<0.001 *
Normal		698 (41.1)	43 (33.1)	
One abnormality		395 (23.2)	20 (15.4)	
Two abnormalities		164 (9.6)	6 (4.6)	
Complex		261 (15.4)	39 (30.0)	
No data		182 (10.7)	22 (16.9)	
ELN classification	1626			<0.001 *
Favorable		449 (26.5)	11 (8.4)	
Intermediate		695 (40.9)	53 (40.8)	
Poor		365 (22.0)	48 (36.9)	
No data		182 (10.7)	22 (16.9)	
Comorbidity	1830			<0.001 *
Others		1679 (98.8)	119 (91.5)	
Renal disease		21 (1.2)	11 (8.5)	

^a^ This variable was not included in the machine learning. Abbreviation: CTx, chemotherapy; Standard, cytarabine and anthracycline; FLAG, fludarabine, cytarabine, granulocyte colony-stimulating factor (GCSF); HMA, hypomethylating agent; LDAC, low dose cytarabine; MEC, mitoxantrone, etoposide, cytarabine. *, statistical significance.

**Table 2:** Laboratory data for day 30 (D30) survivors and non-survivors.

	D30 Survivor	D30 Non-Survivor	*p*
	(*n* = 1700)	(*n* = 130)	
WBC (×10^9^)	35.3 ± 64.0	55.2 ± 85.6	0.010 *
RBC (×10^12^)	2.8 ± 0.6	2.8 ± 0.6	0.463
Hemoglobin (g/dL) ^a^	9.0 ± 1.7	8.7 ± 1.7	0.060
Hematocrit (%) ^a^	26.9 ± 8.3	25.6 ± 4.9	0.008 *
Platelet (×10^9^)	75.5 ± 79.0	75.3 ± 99.5	0.978
Neutrophil (%)	20.1 ± 20.2	16.4 ± 18.5	0.046 *
Lymphocyte (%)	34.2 ± 27.2	26.0 ± 25.5	0.001 *
Monocyte (%)	9.9 ± 15.8	16.1 ± 24.1	0.005 *
Eosinophil (%)	0.7 ± 2.0	0.3 ± 1.3	0.004 *
Basophil (%)	0.2 ± 1.0	0.3 ± 1.1	0.519
Blast (%)	39.0 ± 29.9	41.5 ± 31.7	0.356
ANC (×10^9^) ^a^	3.0 ± 6.7	3.6 ± 6.3	0.318
RDW	16.0 ± 2.0	16.2 ± 2.2	0.192
MCV (fL)	95.5 ± 7.1	92.9 ± 6.8	<0.001 *
MCH (pg) ^a^	32.2 ± 2.6	31.6 ± 2.6	0.012 *
MCHC (%)	33.7 ± 1.3	34.0 ± 1.5	0.016 *
PT (s) ^a^	13.3 ± 1.8	14.7 ± 2.3	<0.001 *
APTT (s)	30.1 ± 6.3	33.3 ± 7.3	<0.001 *
FDP (mcg/mL)	12.6 ± 17.9	24.1 ± 27.0	<0.001 *
Fibrinogen (g/L) ^a^	9.8 ± 2.8	9.36 ± 3.69	0.545
D-dimer (nmol/L)	25.4 ± 41.1	49.2 ± 60.7	<0.001 *
Glucose (mmol/L)	6.8 ± 2.2	7.8 ± 2.9	<0.001 *
BUN (mg/dL)	5.0 ± 2.4	7.2 ± 2.2	<0.001 *
Creatinine (mg/dL)	70.7 ± 35.3	97.2 ± 88.4	0.003
Protein (g/L) ^a^	66.0 ± 7.0	63.0 ± 7.0	<0.001 *
Albumin (g/L)	37.0 ± 5.0	34.0 ± 5.0	<0.001 *
AST (uKat/L)	0.5 ± 0.6	0.8 ± 1.0	0.004 *
ALT (ukat/L)	0.6 ± 0.8	0.7 ± 0.8	0.093
ALP (ukat/L)	1.2 ± 0.8	1.7 ± 1.6	<0.001 *
Bilirubin (umol/L)	11.9 ± 8.5	17.1 ± 11.9	<0.001 *
Direct Bilirubin (umol/L)	5.1 ± 3.4	8.5 ± 10.6	<0.001 *
Uric acid (mmol.L)	0.3 ± 0.1	0.4 ± 0.2	0.002
Calcium (mmol/L)	2.1 ± 0.2	2.1 ± 0.2	0.004
Phosphorus (mmol/L)	1.2 ± 0.3	1.1 ± 0.5	0.453
Sodium (mmol/L)	139.8 ± 3.2	138.3 ± 3.9	<0.001 *
Potassium (mmol/L)	3.9 ± 0.5	3.9 ± 0.5	0.678
Chloride (mmol/L)	103.6 ± 3.4	147.0 ± 6.5	0.330
LDH (ukat/L)	21.9 ± 28.6	37.2 ± 50.4	0.001 *
CPK (ukat/L)	1.4 ± 4.3	1.8 ± 3.3	0.301
Amylase (ukat/L)	1.11 ± 0.7	1.1 ± 0.7	0.440
Magnesium (mmol/L)	0.9 ± 0.1	0.9 ± 0.1	0.494
ESR (mm/h)	33.1 ± 25.7	33.1 ± 26.9	0.999
CRP (mg/L)	42.0 ± 62.0	75.0 ± 78.0	<0.001

^a^ This variable was not included in the machine learning. Abbreviations: WBC, white blood cell; RBC, red blood cell; ANC, absolute neutrophil count; RDW, red cell distribution width; MCV, mean corpuscular volume; MCH, mean cell hemoglobin; MCHC, mean corpuscular hemoglobin concentration; PT, prothrombin time; APTT, activated partial thromboplastin time; FDP, fibrinogen degradation product; BUN, blood urea nitrogen; AST, aspartate aminotransferase; ALT, alanine aminotransferase; ALP, alkaline phosphatase; LDH, lactate dehydrogenase; CPK, creatine phosphokinase; ESR, erythrocyte sedimentation rate; CRP, C-reactive protein. *, statistical significance.

**Table 3:** Result of machine learning for prediction of D30 survival.

	Accuracy	95% CI	Sensitivity	95% CI	Specificity	95% CI
Multivariable LR, initial	89.5	0.873–0.914	3.2	0.004–0.113	95.6	0.941–0.969
Multivariable LR, six features	87.8	0.855–0.899	4.3	0.009–0.122	94.6	0.929–0.961
SVM, initial	93.3	0.915–0.948	0	0.000–0.059	100	0.996–1.000
SVM, six features, un-tuned	91.1	0.801–0.928	14.7	0.070–0.262	96.4	0.111–0.393
SVM, six features, tuned	93.3	0.915–0.948	0	0.000–0.059	100	0.996–1.000
Naïve Bayesian, initial	89.7	0.868–0.911	34.4	0.227–0.477	92.9	0.910–0.946
Naïve Bayesian, six features	91.4	0.893–0.931	37.7	0.256–0.510	95.1	0.935–0.965
Random Forest, initial	94.7	0.931–0.961	26.2	0.158–0.391	99.6	0.990–0.999
Random Forest, six features	93.7	0.920–0.952	39.3	0.271–0.527	97.6	0.964–0.986
Random Forest, six features, tuned	93.3	0.915–0.948	36.1	0.242–0.494	97.4	0.961–0.984
Decision Tree, initial	93.6	0.918–0.951	29.5	0.185–0.426	98.2	0.971–0.990
Decision Tree, six features	90.6	0.885–0.924	70.4	0.885–0.924	92.1	0.885–0.924

Abbreviation: CI, confidence interval; LR, logistic regression; SVM, support vector machine.

## Data Availability

Data are contained within the article or Supplementary Materials.

## Supplementary Materials

The following supporting information can be downloaded at: https://www.mdpi.com/article/10.3390/jcm12185940/s1, Figure S1: Flow chart; Figure S2: Pearson’s correlation matrix for variables; Figure S3: Laboratory data of clinical hematology classified by the 8 prognostic group that showed statistical significance among tested group; Figure S4: Laboratory data of clinical chemistry classified by the 8 prognostic group that showed statistical significance among tested group; Table S1: Comorbidity of study cohort; Table S2: Feature selection analysis results with selected model with 6 variables; Table S3: Variable selected by feature selection analysis; Table S4: Features from various feature selection algorithm; Table S5: Mean survival days of selected variable by feature selection; Table S6: Univariable logistic regression analysis result; Table S7: Multivariable logistic regression analysis result; Table S8; Multivariable logistic regression analysis using 6 variables; Table S9: Positive predictive value (PPV) negative predictive value (NPV) for prediction of D30 survival; Table S10: Clinical and laboratory data for 8 group; Supplementary Table S11: D30 survival days (d) and standard error (SE) for 8 group; Table S12: Pairwise comparison of 8 group by log rank method; Table S13: Prediction of the hemorrhage by multivariable logistic regression analysis by entering statistically significant variables from univariable logistic regression analysis.
