# Supplementary material for: Machine Learning Predicts 30-Day Outcome among Acute Myeloid Leukemia Patients: A Single-Center, Retrospective, Cohort Study
Source: J Clin Med. 2023 Sep 13;12(18):5940. doi: 10.3390/jcm12185940 (PMC10531920; doi:10.3390/jcm12185940)
Supplement: Supplementary file 1 [file jcm-12-05940-s001.zip › Supplementary Materials.docx]

Supplementary Figure S1. Flow chart


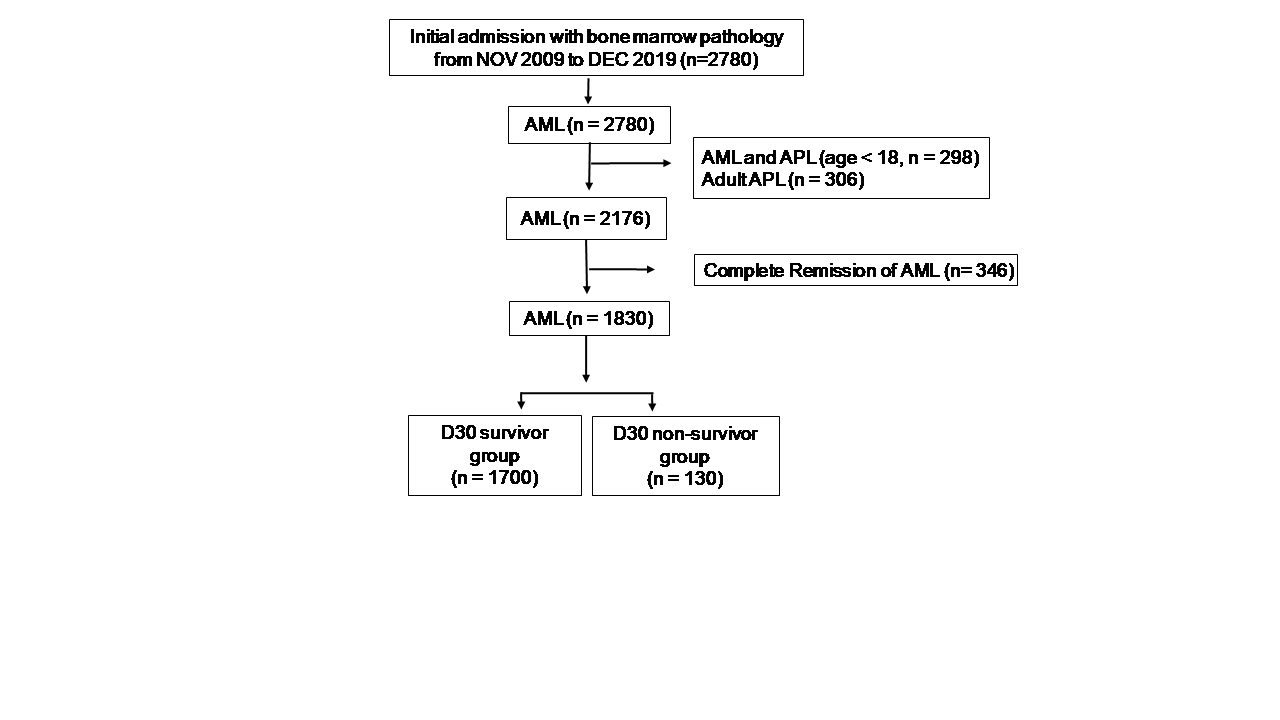


Supplementary Figure S2. Pearson's correlation matrix for variables


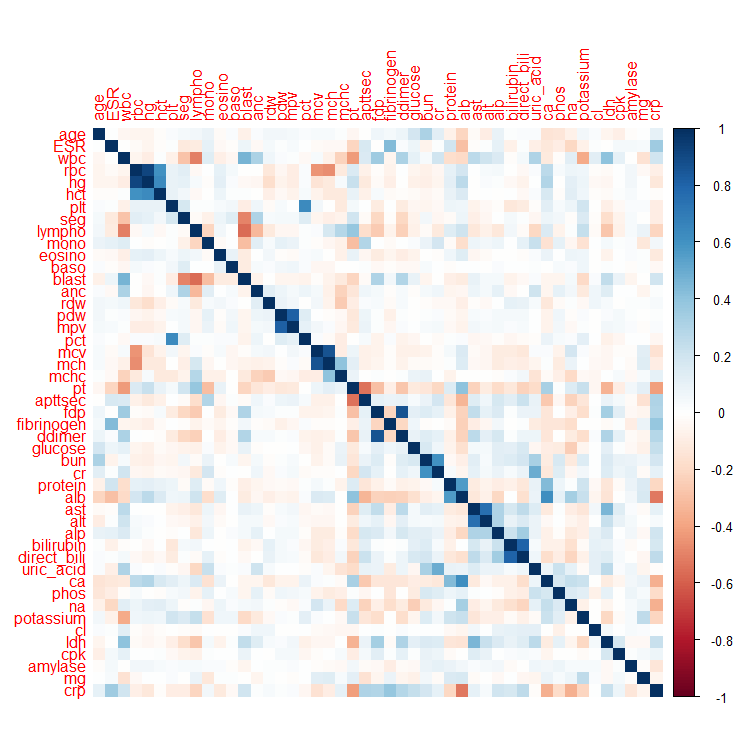


Supplementary Figure S3. Laboratory data of clinical hematology classified by the 8 prognostic group that showed statistical significance among tested group


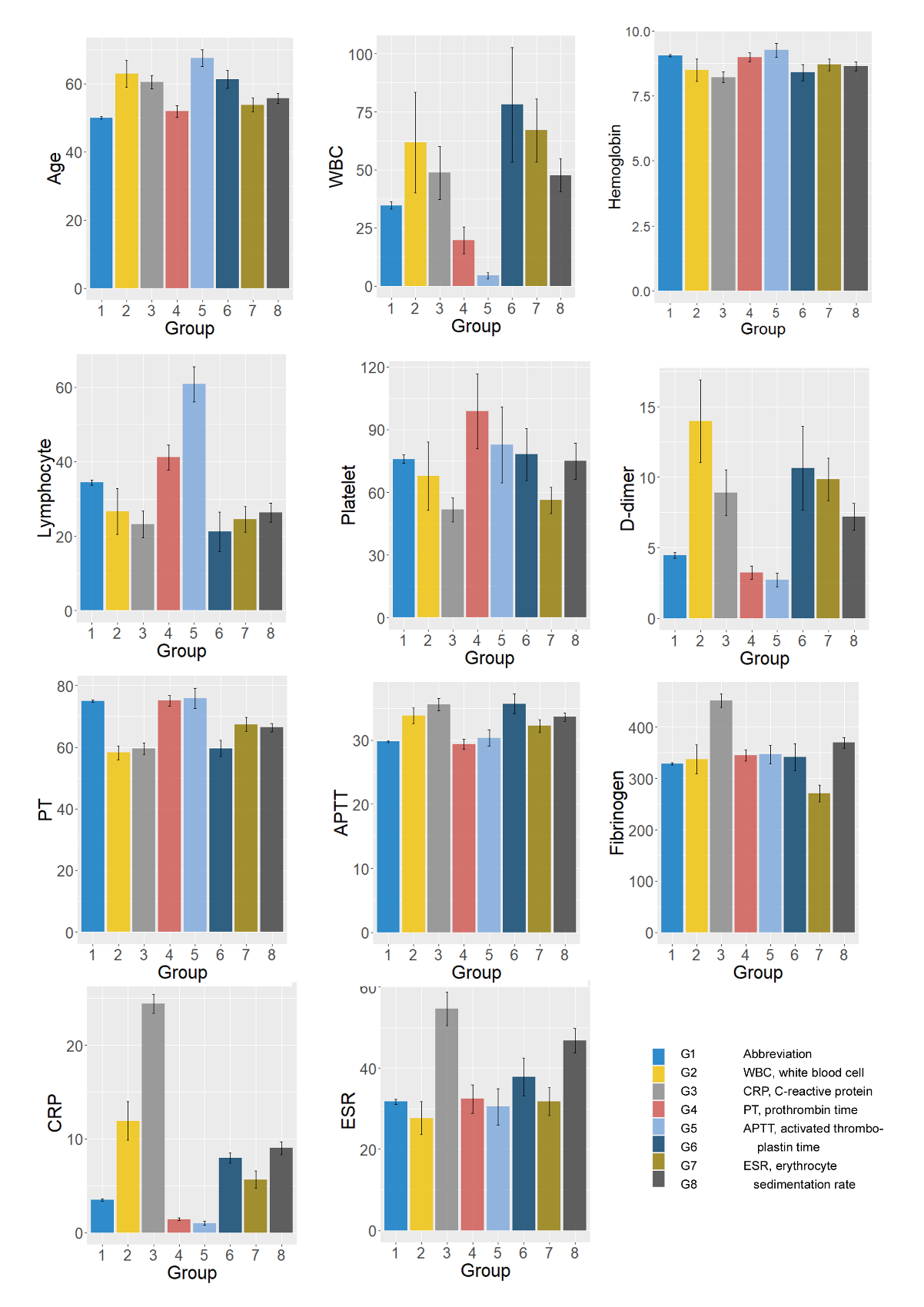


Supplementary Figure S4. Laboratory data of clinical chemistry classified by the 8 prognostic group that showed statistical significance among tested group.


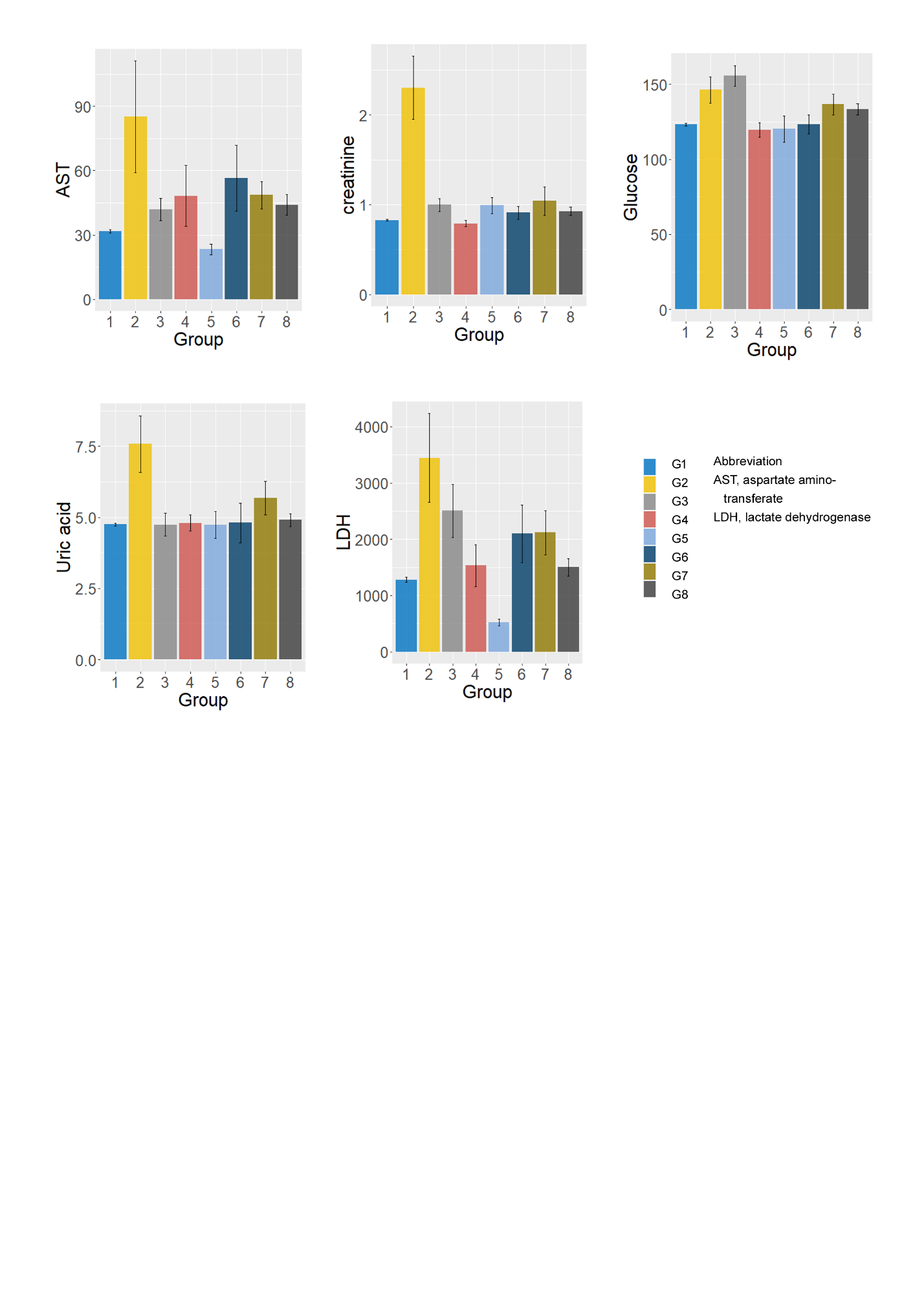


Supplementary Table S1. Comorbidity of study cohort

|  | D30 survivor | D30 non survivor | p |
| --- | --- | --- | --- |
|  | N=1700 (%) | N=130 (%) |  |
| None | 1170 (68.6) | 61 (46.9) | <0.001* |
| Metabolic^a^ | 109 (6.4) | 12 (9.2) |  |
| Cardiac^b^ | 154 (9.1) | 19 (14.6) |  |
| Hepatic | 49 (2.9) | 8 (6.2) |  |
| Renal | 20 (1.2) | 11 (8.5) |  |
| Metabolic, Cardiac | 81 (4.8) | 8 (6.2) |  |
| Metabolic, Hepatic | 1 (0.1) | 0 (0) |  |
| Metabolic, Renal | 1 (0.1) | 1 (0.8) |  |
| Metabolic, Other | 5 (0.3) | 0 (0) |  |
| Cardiac, Hepatic | 4 (0.2) | 0 (0) |  |
| Cardiac, Renal | 2 (0.1) | 0 (0) |  |
| Cardiac, Other | 6 (0.4) | 0 (0) |  |
| Renal, Other disease | 3 (0.2) | 0 (0) |  |
| Metabolic, Cardiac, other | 5 (0.3) | 0 (0) |  |

^a^Metabolic disease are consisted of mainly diabetes mellitus

^b^Cardiac disease are consisted mainly of hypertensive disease

Supplementary Table S2. Feature selection analysis results with selected model with 6 variables

| Variables | Accuracy | Kappa | Accuracy SD | Kappa SD | Selected |
| --- | --- | --- | --- | --- | --- |
| 1 | 0.9247 | 0 | 0.0101 | 0 |  |
| 2 | 0.9302 | 0.1327 | 0.01601 | 0.2137 |  |
| 3 | 0.9269 | 0.101 | 0.01403 | 0.1899 |  |
| 4 | 0.9259 | 0.3056 | 0.02093 | 0.2609 |  |
| 5 | 0.9302 | 0.3481 | 0.02353 | 0.2394 |  |
| 6 | 0.9367 | 0.3573 | 0.01951 | 0.2488 | * |
| 7 | 0.9313 | 0.3122 | 0.02336 | 0.2451 |  |
| 8 | 0.9311 | 0.3055 | 0.01784 | 0.2053 |  |
| 9 | 0.9312 | 0.3495 | 0.0247 | 0.2357 |  |
| 10 | 0.9312 | 0.3437 | 0.02354 | 0.2281 |  |
| 56 | 0.9346 | 0.2227 | 0.01525 | 0.2363 |  |

SD, standard deviation

Supplementary Table S3. Variable selected by feature selection analysis

| Variable | Importance |
| --- | --- |
| Induction | 15.18777 |
| Hemorrhage | 8.459151 |
| Infection | 6.277508 |
| BUN | 5.976411 |
| Direct bilirubin | 5.381942 |
| LDH | 4.520154 |
| PT | 4.488911 |
| AST | 4.389077 |
| ALP | 4.320213 |
| D-dimer | 4.193378 |
| CRP | 4.126103 |
| Karyotype | 3.898549 |
| Age | 3.824017 |
| Platelet | 3.747609 |
| FDP | 3.712098 |
| Amylase | 3.603626 |
| Uric acid | 3.601458 |

Abbreviation: BUN, blood urea nitrogen; LDH, lactate dehydrogenase; PT, prothrombin time; AST, aspartate aminotransferase; ALP, alkaline phosphatase; CRP, C-reactive protein; FDP, fibrinogen degradation product

Supplementary Table S4. Features from various feature selection algorithm

| Random Forest |  | Boruta |  | Xgboost |  | C50 |  |
| --- | --- | --- | --- | --- | --- | --- | --- |
| Variable | Overall |  | Mean Imp |  | Overall |  | Overall |
| Induction | 15.18777 | Induction | 18.874089 | Induction | 100 | Amylase | 100 |
| Hemorrhage | 8.459151 | Hemorrhage | 11.366926 | Hemorrhage | 70.368 | Induction | 100 |
| Infection | 6.277508 | BUN | 8.475769 | Infection | 46.632 | Hemorrhage | 100 |
| BUN | 5.976411 | Direct bilirubin | 6.985677 | PT | 34.1 | BUN | 99.89 |
| Direct bilirubin | 5.381942 | Infection | 6.563263 | BUN | 25.268 | Uric acid | 99.89 |
| L D H | 4.520154 | D-dimer | 5.107776 | MPV | 19.592 | MPV | 99.67 |
| PT | 4.488911 | PT | 4.924907 | CRP | 18.912 | Infection | 99.67 |
| AST | 4.389077 | ALP | 4.049257 | Creatinine | 18.599 | Age | 99.67 |
| ALP | 4.320213 | Age | 4.038034 | MCV | 16.179 | Sodium | 99.34 |
| D-dimer | 4.193378 | L D H | 3.96017 | Glucose | 16.062 | PDW | 99.34 |
| CRP | 4.126103 | Platelet | 3.92828 | Amylase | 15.946 | APTT | 99.23 |
| Cytogenetics | 3.898549 | WBC | 3.716664 | PDW | 14.616 | Creatinine | 98.8 |
| Age | 3.824017 | CRP | 3.694241 | FDP | 11.781 | D-dimer | 98.69 |
| Platelet | 3.747609 | AST | 3.530504 | L D H | 10.202 | MCHC | 98.58 |
| FDP | 3.712098 | Lymphocyte | 3.49593 | Phosphorus | 8.692 | Direct bilirubin | 98.47 |
| Amylase | 3.603626 | FDP | 3.441993 | Direct bilirubin | 8.194 | Platelet | 98.47 |
| Uric acid | 3.601458 | Uric acid | 3.416566 | Platelet | 8.144 | Bilirubin | 98.25 |
| NA |  | Creatinine | 3.264988 | Segment neutrophil | 7.501 | Glucose | 97.92 |
| NA |  | Cytogenetics | 2.544424 | Alkaline phosphatase | 6.492 | MCH | 97.38 |
| NA |  | Comorbidity | 2.495436 | Age | 6.173 | Comorbidity | 97.16 |

Abbreviation: BUN, blood urea nitrogen; LDH, lactate dehydrogenase; PT, prothrombin time; AST, aspartate aminotransferase; ALP, alkaline phosphatase; CRP, C-reactive protein; FDP, fibrinogen degradation product; WBC, white blood cell; MPV, mean platelet volume; MCV, mean corpuscular volume; PDW, platelet distribution width; APTT, activated thromboplastin time; MCH, mean cell hemoglobin

Supplementary Table S5. Mean survival days of selected variables by feature selection

| Variables | records | n.max | n.start | events | rmean | se(rmean) |
| --- | --- | --- | --- | --- | --- | --- |
| Treatment |  |  |  |  |  |  |
| NO CTx | 166 | 166 | 166 | 71 | 22.77711 | 0.73035189 |
| Standard CTx | 1341 | 1341 | 1341 | 34 | 29.79195 | 0.04251837 |
| FLAG | 43 | 43 | 43 | 3 | 29.76744 | 0.15368982 |
| HMA | 133 | 133 | 133 | 13 | 28.97744 | 0.32712663 |
| LDAR | 74 | 74 | 74 | 7 | 29.01351 | 0.39744125 |
| MEC | 66 | 66 | 66 | 2 | 29.68182 | 0.22387264 |
| Others | 7 | 7 | 7 | 0 | 30 | 0 |
| Hemorrhage |  |  |  |  |  |  |
| No | 1775 | 1775 | 1775 | 100 | 29.32394 | 0.07700964 |
| Yes | 55 | 55 | 55 | 30 | 20.58182 | 1.35248554 |
| Infection |  |  |  |  |  |  |
| No | 1562 | 1562 | 1562 | 60 | 29.52113 | 0.07177665 |
| Respiratory | 206 | 206 | 206 | 56 | 26.14078 | 0.51150671 |
| Blood stream | 18 | 18 | 18 | 9 | 24.11111 | 1.72470947 |
| Others | 44 | 44 | 44 | 5 | 28.43182 | 0.75814975 |
| BUN |  |  |  |  |  |  |
| Low | 1245 | 1245 | 1245 | 56 | 29.44819 | 0.084065 |
| High | 585 | 585 | 585 | 74 | 28.23761 | 0.2212865 |
| Direct bilirubin |  |  |  |  |  |  |
| Low | 1215 | 1215 | 1215 | 60 | 29.42469 | 0.08726166 |
| High | 615 | 615 | 615 | 70 | 28.34309 | 0.20934988 |
| L D H |  |  |  |  |  |  |
| Low | 1168 | 1168 | 1168 | 57 | 29.48116 | 0.08087734 |
| High | 662 | 662 | 662 | 73 | 28.32024 | 0.20713109 |

Abbreviation: CTx, chemotherapy; Standard, cytarabine and anthracycline; FLAG, fludarabine, cytarabine, granulocyte colony stimulating factor (GCSF); HMA, hypomethylating agent; LDAC, low dose cytarabine; MEC, mitoxantrone, etoposide, cytarabine; BUN, blood urea nitrogen; LDH, lactate dehydrogenase

Supplementary Table S6. Univariable logistic regression analysis result

| Variable | Estimate | Std. Error | z-value | p-value | significance |
| --- | --- | --- | --- | --- | --- |
| (Intercept) | -2.8120322 | 0.1543482 | -18.218751 | 3.66E-74 |  |
| Age | 0.6353359 | 0.1471389 | 4.317932 | 1.57E-05 | * |
|  |  |  |  |  |  |
| (Intercept) | -2.805882 | 0.2102002 | -13.34862 | 1.21E-40 |  |
| Sex | 0.292117 | 0.270923 | 1.078229 | 2.81E-01 |  |
|  |  |  |  |  |  |
| (Intercept) | -2.6423983 | 0.1329615 | -19.873412 | 6.91E-88 |  |
| ESR | -0.0945672 | 0.1436042 | -0.658527 | 5.10E-01 |  |
|  |  |  |  |  |  |
| (Intercept) | -2.661805 | 0.1350996 | -19.702535 | 2.05E-86 |  |
| WBC | 0.2650932 | 0.1012969 | 2.616993 | 8.87E-03 | * |
|  |  |  |  |  |  |
| (Intercept) | -2.645701 | 0.1334029 | -19.832407 | 1.56E-87 |  |
| RBC | -0.140557 | 0.1384177 | -1.015455 | 3.10E-01 |  |
|  |  |  |  |  |  |
| (Intercept) | -2.6511537 | 0.1341121 | -19.768184 | 5.60E-87 |  |
| Hemoglobin | -0.1901199 | 0.139368 | -1.364158 | 1.73E-01 |  |
|  |  |  |  |  |  |
| (Intercept) | -2.6685981 | 0.1364405 | -19.558694 | 3.48E-85 |  |
| Hematocrit | -0.4016337 | 0.2186207 | -1.837126 | 6.62E-02 | * |
|  |  |  |  |  |  |
| (Intercept) | -2.7424962 | 0.1502845 | -18.248692 | 2.12E-74 |  |
| PLT | -0.6409252 | 0.2694388 | -2.378741 | 1.74E-02 | * |
|  |  |  |  |  |  |
| (Intercept) | -2.6476482 | 0.1340464 | -19.751728 | 7.75E-87 |  |
| Segmented neutrophil | -0.2326338 | 0.1469425 | -1.583163 | 1.13E-01 |  |
|  |  |  |  |  |  |
| (Intercept) | -2.6674113 | 0.1362765 | -19.573519 | 2.60E-85 |  |
| Lymphocyte | -0.2736811 | 0.1494992 | -1.830653 | 6.72E-02 |  |
|  |  |  |  |  |  |
| (Intercept) | -2.6842664 | 0.1371455 | -19.572398 | 2.66E-85 |  |
| Monocyte | 0.2995553 | 0.1008842 | 2.969297 | 2.98E-03 |  |
|  |  |  |  |  |  |
| (Intercept) | -2.6730782 | 0.1387269 | -19.26863 | 9.85E-83 |  |
| Eosinophil | -0.3635686 | 0.2812358 | -1.292753 | 1.96E-01 |  |
|  |  |  |  |  |  |
| (Intercept) | -2.6436332 | 0.13299265 | -19.87804 | 6.30E-88 |  |
| Basophil | 0.084378 | 0.09514124 | 0.8868707 | 3.75E-01 |  |
|  |  |  |  |  |  |
| (Intercept) | -2.6383575 | 0.1325366 | -19.90664 | 3.56E-88 |  |
| Blast | 0.0390313 | 0.1362489 | 0.2864703 | 7.75E-01 |  |
|  |  |  |  |  |  |
| (Intercept) | -2.6404398 | 0.132711 | -19.896169 | 4.39E-88 |  |
| ANC | 0.0311263 | 0.1085515 | 0.2867424 | 7.74E-01 |  |
|  |  |  |  |  |  |
| (Intercept) | -2.639061 | 0.1325324 | -19.912577 | 3.17E-88 |  |
| RDW | 0.0105697 | 0.1326154 | 0.07970211 | 9.36E-01 |  |
|  |  |  |  |  |  |
| (Intercept) | -2.6422442 | 0.1330528 | -19.858615 | 9.28E-88 |  |
| PDW | 0.1406753 | 0.1153859 | 1.219172 | 2.23E-01 |  |
|  |  |  |  |  |  |
| (Intercept) | -2.6461777 | 0.1334854 | -19.823723 | 1.86E-87 |  |
| MPV | 0.1728026 | 0.1209255 | 1.429001 | 1.53E-01 |  |
|  |  |  |  |  |  |
| (Intercept) | -2.6412457 | 0.1327987 | -19.889099 | 5.06E-88 |  |
| PCT | 0.0423887 | 0.1096733 | 0.3864993 | 6.99E-01 |  |
|  |  |  |  |  |  |
| (Intercept) | -2.6849104 | 0.1382362 | -19.422628 | 4.97E-84 |  |
| MCV | -0.3371276 | 0.1402251 | -2.404189 | 1.62E-02 | * |
|  |  |  |  |  |  |
| (Intercept) | -2.6427869 | 0.1330039 | -19.869991 | 7.40E-88 |  |
| MCH | -0.1053331 | 0.135383 | -0.7780379 | 4.37E-01 |  |
|  |  |  |  |  |  |
| (Intercept) | -2.7049551 | 0.1401152 | -19.30522 | 4.85E-83 |  |
| MCHC | 0.3816253 | 0.1380964 | 2.76347 | 5.72E-03 | * |
|  |  |  |  |  |  |
| (Intercept) | -2.8781733 | 0.1603107 | -17.953717 | 4.49E-72 |  |
| PT | -0.7871741 | 0.1406337 | -5.597336 | 2.18E-08 | * |
|  |  |  |  |  |  |
| (Intercept) | -2.7076846 | 0.1390726 | -19.46958 | 1.99E-84 |  |
| APTT | 0.3819929 | 0.1099549 | 3.474086 | 5.13E-04 | * |
|  |  |  |  |  |  |
| (Intercept) | -2.72648 | 0.14151086 | -19.266932 | 1.02E-82 |  |
| FDP | 0.4559953 | 0.09423468 | 4.838933 | 1.31E-06 | * |
|  |  |  |  |  |  |
| (Intercept) | -2.679213 | 0.1375487 | -19.478283 | 1.68E-84 |  |
| Fibrinogen | -0.3216769 | 0.1362107 | -2.361613 | 1.82E-02 | * |
|  |  |  |  |  |  |
| (Intercept) | -2.7186708 | 0.140528 | -19.346114 | 2.20E-83 |  |
| D-dimer | 0.4203255 | 0.09295434 | 4.521849 | 6.13E-06 | * |
|  |  |  |  |  |  |
| (Intercept) | -2.6522679 | 0.1340254 | -19.7893 | 3.68E-87 |  |
| Glucose | 0.2066372 | 0.1054647 | 1.959302 | 5.01E-02 | * |
|  |  |  |  |  |  |
| (Intercept) | -2.7304782 | 0.14079349 | -19.393497 | 8.76E-84 |  |
| BUN | 0.4380061 | 0.09236281 | 4.742235 | 2.11E-06 | * |
|  |  |  |  |  |  |
| (Intercept) | -2.683499 | 0.1366711 | -19.634726 | 7.81E-86 |  |
| Creatinine | 0.3764493 | 0.114652 | 3.283407 | 1.03E-03 | * |
|  |  |  |  |  |  |
| (Intercept) | -2.68721 | 0.1387478 | -19.36759 | 1.45E-83 |  |
| Protein | -0.41868 | 0.139681 | -2.9974 | 2.72E-03 | * |
|  |  |  |  |  |  |
| (Intercept) | -2.753658 | 0.1463283 | -18.818356 | 5.34E-79 |  |
| ALB | -0.5408702 | 0.1352404 | -3.999323 | 6.35E-05 | * |
|  |  |  |  |  |  |
| (Intercept) | -2.671619 | 0.13532927 | -19.741619 | 9.47E-87 |  |
| AST | 0.261318 | 0.07977431 | 3.275716 | 1.05E-03 | * |
|  |  |  |  |  |  |
| (Intercept) | -2.6479275 | 0.1334417 | -19.843323 | 1.26E-87 |  |
| ALT | 0.1542876 | 0.1004876 | 1.535388 | 1.25E-01 |  |
|  |  |  |  |  |  |
| (Intercept) | -2.6972654 | 0.13788272 | -19.56203 | 3.26E-85 |  |
| ALP | 0.3558633 | 0.08766078 | 4.05955 | 4.92E-05 | * |
|  |  |  |  |  |  |
| (Intercept) | -2.7147547 | 0.13952208 | -19.457528 | 2.52E-84 |  |
| bilirubin | 0.3706255 | 0.09608781 | 3.857154 | 1.15E-04 | * |
|  |  |  |  |  |  |
| (Intercept) | -2.7257174 | 0.13965069 | -19.51811 | 7.70E-85 |  |
| Direct bilirubin | 0.3780104 | 0.08294443 | 4.557394 | 5.18E-06 | * |
|  |  |  |  |  |  |
| (Intercept) | -2.6886909 | 0.1379188 | -19.494736 | 1.22E-84 |  |
| Uric acid | 0.3834195 | 0.1216506 | 3.151811 | 1.62E-03 | * |
|  |  |  |  |  |  |
| (Intercept) | -2.6692113 | 0.13622 | -19.59486 | 1.71E-85 |  |
| Ca | -0.2776128 | 0.1283863 | -2.162323 | 3.06E-02 | * |
|  |  |  |  |  |  |
| (Intercept) | -2.6389579 | 0.132532 | -19.911859 | 3.21E-88 |  |
| Phos | 0.0112135 | 0.133462 | 0.08402048 | 9.33E-01 |  |
|  |  |  |  |  |  |
| (Intercept) | -2.695044 | 0.1385941 | -19.445581 | 3.18E-84 |  |
| Na | -0.340879 | 0.1160554 | -2.937208 | 3.31E-03 | * |
|  |  |  |  |  |  |
| (Intercept) | -2.6567852 | 0.1346696 | -19.728167 | 1.24E-86 |  |
| Potassium | -0.2175389 | 0.1305707 | -1.666062 | 9.57E-02 | * |
|  |  |  |  |  |  |
| (Intercept) | -2.805799 | 0.1889132 | -14.852318 | 6.72E-50 |  |
| Cl | -6.606601 | 4.94156 | -1.336946 | 1.81E-01 |  |
|  |  |  |  |  |  |
| (Intercept) | -2.6610844 | 0.13477377 | -19.744825 | 8.89E-87 |  |
| LDH | 0.2701698 | 0.09368842 | 2.883706 | 3.93E-03 | * |
|  |  |  |  |  |  |
| (Intercept) | -2.638962 | 0.1326 | -19.901679 | 3.94E-88 |  |
| CPK | 0.0038927 | 0.1819062 | 0.02139933 | 9.83E-01 |  |
|  |  |  |  |  |  |
| (Intercept) | -2.6599422 | 0.1356466 | -19.609347 | 1.29E-85 |  |
| Amylase | -0.2508949 | 0.2058359 | -1.218907 | 2.23E-01 |  |
|  |  |  |  |  |  |
| (Intercept) | -2.6406674 | 0.132817 | -19.882006 | 5.83E-88 |  |
| Mg | -0.1087829 | 0.1309563 | -0.8306813 | 4.06E-01 |  |
|  |  |  |  |  |  |
| (Intercept) | -2.682595 | 0.1371152 | -19.564541 | 3.10E-85 |  |
| CRP | 0.311177 | 0.1005928 | 3.093431 | 1.98E-03 | * |
|  |  |  |  |  |  |
| (Intercept) | -2.8019163 | 0.2247426 | -12.467226 | 1.13E-35 |  |
| Karyotype, normal | -0.1280646 | 0.3824561 | -0.3348477 | 7.38E-01 |  |
| Karyotype, 1 abnormalities | -1.6407349 | 1.0306647 | -1.5919193 | 1.11E-01 |  |
| Karyotype, 2 abnormalities | 0.9021682 | 0.3331889 | 2.7076783 | 6.78E-03 | * |
| Karyotype, no data | 0.4993312 | 0.4156097 | 1.2014426 | 2.30E-01 |  |
|  |  |  |  |  |  |
| (Intercept) | -3.3712667 | 0.1994555 | -16.902352 | 4.32E-64 | * |
| Infection, respiratory | 2.5445881 | 0.3018638 | 8.429589 | 3.47E-17 | * |
| Infection, blood stream | 2.9012631 | 0.6039723 | 4.803636 | 1.56E-06 | * |
| Infection, others | 0.8455381 | 0.7614345 | 1.110454 | 2.67E-01 |  |
|  |  |  |  |  |  |
| (Intercept) | -0.3654598 | 0.2232038 | -1.6373368 | 1.02E-01 |  |
| Induction, Standard CTx | -3.4801181 | 0.3503929 | -9.9320439 | 3.02E-23 | * |
| Induction, FLAG CTx | -2.8125941 | 1.0447424 | -2.6921413 | 7.10E-03 | * |
| Induction, HMA | -1.5440827 | 0.4397027 | -3.5116519 | 4.45E-04 | * |
| Induction, LDAC | -2.1194469 | 0.641039 | -3.3062681 | 9.45E-04 | * |
| Induction, MEC | -3.1002761 | 1.0397451 | -2.9817655 | 2.87E-03 | * |
| Induction, others | -15.200609 | 727.6988004 | -0.0208886 | 9.83E-01 |  |
|  |  |  |  |  |  |
| (Intercept) | -2.909511 | 0.1514007 | -19.217289 | 2.65E-82 |  |
| Hemorrhage | 3.314976 | 0.435418 | 7.613319 | 2.67E-14 | * |
|  |  |  |  |  |  |
| (Intercept) | -2.726069 | 0.1391833 | -19.586176 | 2.03E-85 |  |
| Comorbidity, renal disease | 1.878771 | 0.5074123 | 3.702651 | 2.13E-04 | * |

Abbreviations: WBC, white blood cell; RBC, red blood cell; ANC, absolute neutrophil count; RDW, red cell distribution width; MCV, mean corpuscular volume; MCH, mean cell hemoglobin; MCHC, mean corpuscular hemoglobin concentration; PT, prothrombin time; aPTT, activated partial thromboplastin time; FDP, fibrinogen degradation product; BUN, blood urea nitrogen; AST, aspartate aminotransferase; ALT, alanine aminotransferase; ALP, alkaline phosphatase; LDH, lactate dehydrogenase; CPK, creatine phosphokinase; ESR, esrythrocyte sedimentation rate; CRP, C-reactive protein

Supplementary Table S7. Multivariable logistic regression analysis result

| Variable | Estimate | Std. Error | z-value | p-value | significance |
| --- | --- | --- | --- | --- | --- |
| Age | 0.35578 | 0.24022 | 1.481 | 0.1386 |  |
| WBC | 0.29472 | 0.22683 | 1.299 | 0.193838 |  |
| Hematocrit | -0.02167 | 0.3172 | -0.068 | 0.945525 |  |
| PLT | 0.19941 | 0.13153 | 1.516 | 0.129503 |  |
| MCV | -0.30924 | 0.19468 | -1.588 | 0.112175 |  |
| MCHC | 0.11682 | 0.1877 | 0.622 | 0.533707 |  |
| PT | -0.36258 | 0.2775 | -1.307 | 0.191345 |  |
| APTT | -0.04899 | 0.2316 | -0.212 | 0.832471 |  |
| FDJP | 0.04915 | 0.29917 | 0.164 | 0.869498 |  |
| D-dimer | 0.11993 | 0.2736 | 0.438 | 0.66115 |  |
| Fibrinogen | 0.20668 | 0.18982 | 1.089 | 0.276245 |  |
| BUN | 0.14574 | 0.18142 | 0.803 | 0.421779 |  |
| Creatinine | 0.06615 | 0.18526 | 0.357 | 0.721037 |  |
| Glucose | 0.24466 | 0.15679 | 1.56 | 0.118662 |  |
| Protein | -0.31006 | 0.23322 | -1.329 | 0.183684 |  |
| Albumin | 0.33616 | 0.32413 | 1.037 | 0.299674 |  |
| AST | -0.22642 | 0.21423 | -1.057 | 0.29056 |  |
| ALP | 0.13846 | 0.14935 | 0.927 | 0.353871 |  |
| Bilirubin | -0.99498 | 0.45601 | -2.182 | 0.029115 | * |
| Direct bilirubin | 1.1469 | 0.45381 | 2.527 | 0.011494 | * |
| Uric acid | 0.16931 | 0.17219 | 0.983 | 0.32547 |  |
| Ca | 0.22069 | 0.2214 | 0.997 | 0.318878 |  |
| Na | -0.0772 | 0.20344 | -0.379 | 0.704344 |  |
| Potassium | 0.11317 | 0.18079 | 0.626 | 0.531324 |  |
| CRP | -0.10616 | 0.20839 | -0.509 | 0.610449 |  |
| ELN, favorable | -0.52718 | 0.59317 | -0.889 | 0.374139 |  |
| ELN, intermediate | 0.46289 | 0.70751 | 0.654 | 0.51295 |  |
| ELN, poor | 0.16231 | 0.58705 | 0.276 | 0.782172 |  |
| ELN, no data | 0.59918 | 0.55875 | 1.072 | 0.283554 |  |
| Infection, respiratory | 1.51098 | 0.42334 | 3.569 | 3.58E-04 | * |
| Infection, blood stream | 4.11293 | 1.39446 | 2.949 | 0.003183 | * |
| Infection, others | 1.57276 | 0.85396 | 1.842 | 0.065516 |  |
| Hemorrhage | 3.10721 | 0.5939 | 5.232 | 1.68E-07 | * |
| Comorbidity, renal disease | 1.24247 | 0.95724 | 1.298 | 0.194301 |  |
| Induction, Standard CTx | -2.12051 | 0.48328 | -4.388 | 1.15E-05 | * |
| Induction, FLAG CTx | -0.16392 | 1.02087 | -0.161 | 0.872432 |  |
| Induction, HMA | -2.08826 | 0.64979 | -3.214 | 0.00131 | * |
| Induction, LDAC | -2.30673 | 0.86043 | -2.681 | 0.007343 | * |
| Induction, MEC | -2.10167 | 1.32981 | -1.58 | 0.114009 |  |
| Induction, others | -12.4598 | 816.49464 | -0.015 | 0.987825 |  |

Abbreviations: WBC, white blood cell; RBC, red blood cell; ANC, absolute neutrophil count; RDW, red cell distribution width; MCV, mean corpuscular volume; MCH, mean cell hemoglobin; MCHC, mean corpuscular hemoglobin concentration; PT, prothrombin time; aPTT, activated partial thromboplastin time; FDP, fibrinogen degradation product; BUN, blood urea nitrogen; AST, aspartate aminotransferase; ALT, alanine aminotransferase; ALP, alkaline phosphatase; LDH, lactate dehydrogenase; CPK, creatine phosphokinase; ESR, erythrocyte sedimentation rate; CRP, C-reactive protein

Supplementary Table S8. Multivariable logistic regression analysis using 6 variables

|  | OR | lcl | ucl | p |
| --- | --- | --- | --- | --- |
| (Intercept) | 0.23 | 0.12 | 0.43 | <0.001 |
| Hemorrhage | 16.69 | 6.49 | 42.9 | <0.001 |
| Induction, Standard CTx | 0.07 | 0.03 | 0.14 | <0.001 |
| Induction, FLAG | 0.5 | 0.1 | 2.53 | 0.4056 |
| Induction, HMA | 0.14 | 0.05 | 0.42 | 0.0005 |
| Induction, LDAG | 0.15 | 0.04 | 0.53 | 0.0032 |
| Induction, MEC | 0.1 | 0.01 | 0.85 | 0.0351 |
| Induction, Others | 0 | 0 | Inf | 0.9868 |
| Infection, respiratory | 4.22 | 2.11 | 8.44 | <0.001 |
| Infection, blood stream | 90.55 | 6.62 | 1238.19 | 0.0007 |
| Infection, others | 3.75 | 0.83 | 16.89 | 0.0849 |
| CRP | 1.05 | 0.79 | 1.39 | 0.7429 |
| BUN | 1.4 | 1.11 | 1.79 | 0.0055 |
| L D H | 1.19 | 0.95 | 1.48 | 0.1348 |

Abbreviation: CTx, chemotherapy; Standard, cytarabine and anthracycline; FLAG, fludarabine, cytarabine, granulocyte colony stimulating factor (GCSF); HMA, hypomethylating agent; LDAC, low dose cytarabine; MEC, mitoxantrone, etoposide, cytarabine; CRP, C-reactive protein; BUN, blood urea nitrogen; LDH, lactate dehydrogenase

Supplementary Table S9. Positive predictive value (PPV) and negative predictive value (NPV) of for prediction of D30 survival

|  | PPV | 95%CI | NPV | 95%CI |
| --- | --- | --- | --- | --- |
| Multivariable LR, initial | 5.1 | 0.006-0.173 | 93.2 | 0.914-0.948 |
| Multivariable LR, 6 feature | 6.2 | 0.013-0.172 | 92.3 | 0.904-0.941 |
|  |  |  |  |  |
| SVM, initial | 0 | 0 | 99.3 | 0.915-0.949 |
| SVM, 6 features, un-tuned | 23.1 | 0.950-0.976 | 96.4 | 0.923-0.955 |
| SVM, 6 features, tuned | 0 | 0 | 93.3 | 0.915-0.949 |
|  |  |  |  |  |
| Naïve Bayesian, initial | 25.9 | 0.168-0.369 | 95.2 | 0.935-0.966 |
| Naïve Bayesian, 6 features | 35.9 | 0.243-0.489 | 95.5 | 0.939-0.968 |
|  |  |  |  |  |
| Random Forest, initial | 84.2 | 0.604-0.966 | 94.9 | 0.933-0.963 |
| Random Forest, 6 features | 54.4 | 0.304-0.612 | 95.7 | 0.942-0.970 |
| Random Forest, 6 features, tuned | 50.1 | 0.346-0.654 | 95.5 | 0.939-0.968 |
|  |  |  |  |  |
| Decision Tree, initial | 54.5 | 0.364-0.719 | 95.1 | 0.935-0.964 |
| Decision Tree, 6 features | 38.7 | 0.296-0.485 | 97.7 | 0.965-0.987 |

Abbreviation: PPV, positive predictive value; NPV, negative predictive value; CI, confidence interval; LR, logistic regression; SVM, support vector machine

Supplementary Table S11. D30 survival days (d) and standard error (SE) for 8 group.

| Group | Patient | Death | Mean (d) | SE (d) |
| --- | --- | --- | --- | --- |
| G1 | 1502 | 33 | 29.8 | 0.0 |
| G2 | 20 | 6 | 26.6 | 1.5 |
| G3 | 41 | 9 | 26.4 | 1.1 |
| G4 | 64 | 14 | 27.0 | 0.8 |
| G5 | 20 | 4 | 28.8 | 0.6 |
| G6 | 22 | 10 | 23.7 | 1.7 |
| G7 | 55 | 30 | 20.6 | 1.4 |
| G8 | 106 | 24 | 27.2 | 0.6 |

Supplementary Table S12. Pairwise comparison of 8 group by log rank method

|  | G1 | G2 | G3 | G4 | G5 | G6 | G7 |
| --- | --- | --- | --- | --- | --- | --- | --- |
| G2 | 2.50E-16 | - | - | - | - | - | - |
| G3 | 4.60E-16 | 0.74736 | - | - | - | - | - |
| G4 | < 2e-16 | 0.6685 | 0.97831 | - | - | - | - |
| G5 | 6.30E-07 | 0.6216 | 0.8473 | 0.88895 | - | - | - |
| G6 | < 2e-16 | 0.436 | 0.11517 | 0.05547 | 0.1035 | - | - |
| G7 | < 2e-16 | 0.09082 | 0.00377 | 0.00028 | 0.01523 | 0.52875 | - |
| G8 | < 2e-16 | 0.67854 | 0.97831 | 0.97831 | 0.8473 | 0.04179 | 1.80E-05 |

Supplementary Table S13. Prediction of the hemorrhage by multivariable logistic regression analysis by entering statistically significant variables from univariable logistic regression analysis

|  | OR | Lower CI | Upper CI | p-value |
| --- | --- | --- | --- | --- |
| (Intercept) | 0.02 | 0.01 | 0.04 | <0.001 |
| Age | 0.84 | 0.55 | 1.29 | 0.4217 |
| WBC | 0.87 | 0.56 | 1.35 | 0.5418 |
| Lymphocyte | 0.79 | 0.49 | 1.27 | 0.3282 |
| Monocyte | 0.87 | 0.56 | 1.37 | 0.5595 |
| PT | 0.98 | 0.62 | 1.55 | 0.9336 |
| FDP | 1.3 | 0.94 | 1.81 | 0.1105 |
| BUN | 1.48 | 1.06 | 2.06 | 0.0197 |
| Albumin | 1.06 | 0.67 | 1.67 | 0.8091 |
| AST | 0.5 | 0.19 | 1.32 | 0.1616 |
| ALT | 1.46 | 0.75 | 2.83 | 0.2627 |
| Fibrinogen | 0.61 | 0.41 | 0.92 | 0.0182 |
| ALP | 1.32 | 1 | 1.75 | 0.049 |
| Uric acid | 0.89 | 0.63 | 1.26 | 0.5231 |
| Phosphorus | 0.62 | 0.41 | 0.93 | 0.0202 |
| Potassium | 0.75 | 0.49 | 1.15 | 0.1849 |
| L D H | 1.1 | 0.68 | 1.78 | 0.7034 |

Abbreviation: WBC, white blood cell; PT, prothrombin time; FDP, fibrinogen degradation product; BUN, blood urea nitrogen; AST, aspartate aminotransferase; ALT, alanine aminotransferase; ALP, alkaline phosphatase; LDH, lactate dehydrogenase
